# A Comprehensive Youth Diabetes Epidemiological Data Set and Web Portal: Resource Development and Case Studies

**DOI:** 10.2196/53330

**Published:** 2024-07-02

**Authors:** Catherine McDonough, Yan Chak Li, Nita Vangeepuram, Bian Liu, Gaurav Pandey

**Affiliations:** 1 Department of Genetics and Genomic Sciences Icahn School of Medicine at Mount Sinai New York, NY United States; 2 Department of Pediatrics Icahn School of Medicine at Mount Sinai New York, NY United States; 3 Department of Population Health Science and Policy Icahn School of Medicine at Mount Sinai New York, NY United States

**Keywords:** youth prediabetes and diabetes, public data set, NHANES, web portal, epidemiology, biostatistics, machine learning, National Health and Nutrition Examination Survey

## Abstract

**Background:**

The prevalence of type 2 diabetes mellitus (DM) and pre–diabetes mellitus (pre-DM) has been increasing among youth in recent decades in the United States, prompting an urgent need for understanding and identifying their associated risk factors. Such efforts, however, have been hindered by the lack of easily accessible youth pre-DM/DM data.

**Objective:**

We aimed to first build a high-quality, comprehensive epidemiological data set focused on youth pre-DM/DM. Subsequently, we aimed to make these data accessible by creating a user-friendly web portal to share them and the corresponding codes. Through this, we hope to address this significant gap and facilitate youth pre-DM/DM research.

**Methods:**

Building on data from the National Health and Nutrition Examination Survey (NHANES) from 1999 to 2018, we cleaned and harmonized hundreds of variables relevant to pre-DM/DM (fasting plasma glucose level ≥100 mg/dL or glycated hemoglobin  ≥5.7%) for youth aged 12-19 years (N=15,149). We identified individual factors associated with pre-DM/DM risk using bivariate statistical analyses and predicted pre-DM/DM status using our Ensemble Integration (EI) framework for multidomain machine learning. We then developed a user-friendly web portal named Prediabetes/diabetes in youth Online Dashboard (POND) to share the data and codes.

**Results:**

We extracted 95 variables potentially relevant to pre-DM/DM risk organized into 4 domains (sociodemographic, health status, diet, and other lifestyle behaviors). The bivariate analyses identified 27 significant correlates of pre-DM/DM (*P*<.001, Bonferroni adjusted), including race or ethnicity, health insurance, BMI, added sugar intake, and screen time. Among these factors, 16 factors were also identified based on the EI methodology (Fisher *P* of overlap=7.06×10^-6^). In addition to those, the EI approach identified 11 additional predictive variables, including some known (eg, meat and fruit intake and family income) and less recognized factors (eg, number of rooms in homes). The factors identified in both analyses spanned across all 4 of the domains mentioned. These data and results, as well as other exploratory tools, can be accessed on POND.

**Conclusions:**

Using NHANES data, we built one of the largest public epidemiological data sets for studying youth pre-DM/DM and identified potential risk factors using complementary analytical approaches. Our results align with the multifactorial nature of pre-DM/DM with correlates across several domains. Also, our data-sharing platform, POND, facilitates a wide range of applications to inform future youth pre-DM/DM studies.

## Introduction

Type 2 diabetes mellitus (DM) is a complex disease influenced by several biological and epidemiological factors [1,2], such as obesity [3], family history [4], diet [1,5], physical activity level [1,6-8], and socioeconomic status [9-11]. Prediabetes, characterized by elevated blood glucose levels below the diabetes threshold, is a precursor condition to DM [12]. There has been an alarming increasing trend in the prevalence of youth with pre–diabetes mellitus (pre-DM) and DM both in the United States [13-19] and worldwide [20,21], and the numbers of newly diagnosed youth living with pre-DM/DM are also expected to increase [14,20,22]. The latest estimate based on nationally representative data showed that the prevalence of pre-DM among youth increased from 11.6% in 1999-2002 to 28.2% in 2015-2018 in the United States [13]. This growth is particularly concerning because pre-DM/DM disproportionately affects racial and ethnic minority groups and those with low socioeconomic status [9-11,22-24], leading to significant health disparities. Having pre-DM/DM at a younger age also confers a higher health and economic burden resulting from living with the condition for more years and a higher risk of developing other cardiometabolic diseases [25-30]. This serious challenge calls for increased translational research into factors associated with pre-DM/DM among youth and how they can collectively affect disease risk and inform prevention strategies.

In particular, the most critically needed research in this direction is exploring the collective impact of various risk factors across multiple health-related domains. While clinical factors, such as obesity, have been mechanistically linked to insulin resistance [31], it is important to consider the broader perspective. There is an increasing recognition that social determinants of health (SDoH) play a significant role in amplifying the risk of pre-DM/DM and their related disparities. For example, factors such as limited access to health care, food and housing insecurity, and the neighborhood-built environment have been identified as influential contributors [9-11,32]. However, to gain a comprehensive understanding, it is essential to delve into other less studied variables, such as screen time, acculturation, or frequency of eating out, and examine how they interact to increase the risk of pre-DM/DM among youth [2].

One of the major challenges that has limited translational research into youth pre-DM/DM risk factors is that there are not publicly available, easily accessible data comprehensively profiling interrelated epidemiological factors for young individuals [2]. Specifically, most available public diabetes data portals focus on providing aggregated descriptive trends, such as pre-DM/DM prevalence for the entire population or subgroups stratified by race and ethnicity [33-36], which does not allow in-depth examination of the relationships between multiple risk factors and pre-DM/DM risk using individual-level data. While there do exist a few individual-level public diabetes data sets [37-41], they include mainly clinical measurements, while other important risk factors such as those related to diet, physical activity, and SDoH are limited. In addition, these data sets are not available for youth populations, as they focus exclusively on adult populations and not on youth specifically [37,39-41]. Furthermore, these data sets are not accompanied by any user-friendly web-based portals that can help explore or analyze these data to reveal interesting knowledge about youth pre-DM/DM. This shows that there is a lack of a comprehensive data set that includes multiple epidemiological variables to study youth pre-DM/DM and easily usable functionalities to explore and analyze data.

To directly address this data gap, we turned to the National Health and Nutrition Examination Survey (NHANES), which offers a promising path for examining pre-DM/DM among the US youth population by providing a rich source of individual- and household-level epidemiological factors. As a result, NHANES has been a prominent data source for studying youth pre-DM/DM trends and associated factors [18,42-45]. However, the use of NHANES data requires extensive data processing that is laborious and time-intensive [46]. This represents a major challenge for the widespread use of these high-quality and extensive data for studying youth pre-DM/DM.

In this work, we directly addressed the above challenges by processing NHANES data from 1999 to 2018 into a large-scale, youth diabetes–focused data set that covers a variety of relevant variable domains, namely, sociodemographic factors, health status indicators, diet, and other lifestyle behaviors. We also provided public access to this high-quality comprehensive youth pre-DM/DM data set, as well as functionalities to explore and analyze it, through the user-friendly Prediabetes/diabetes in youth Online Dashboard (POND) [47]. We demonstrated the data set’s use and potential through 2 case studies that used statistical analyses and machine learning (ML) approaches, respectively, to identify important epidemiological factors that are associated with youth pre-DM/DM.

Through this work, we aim to advance youth diabetes research by providing the most comprehensive epidemiological data set available through a public web portal and illustrating the value of these resources through our example case studies based on statistical analyses and ML. Our overarching goal is to enable researchers to investigate the multifactorial variables associated with youth pre-DM/DM, which may drive translational advances in prevention and management strategies.

## Methods

### Overview

**Figure 1:**
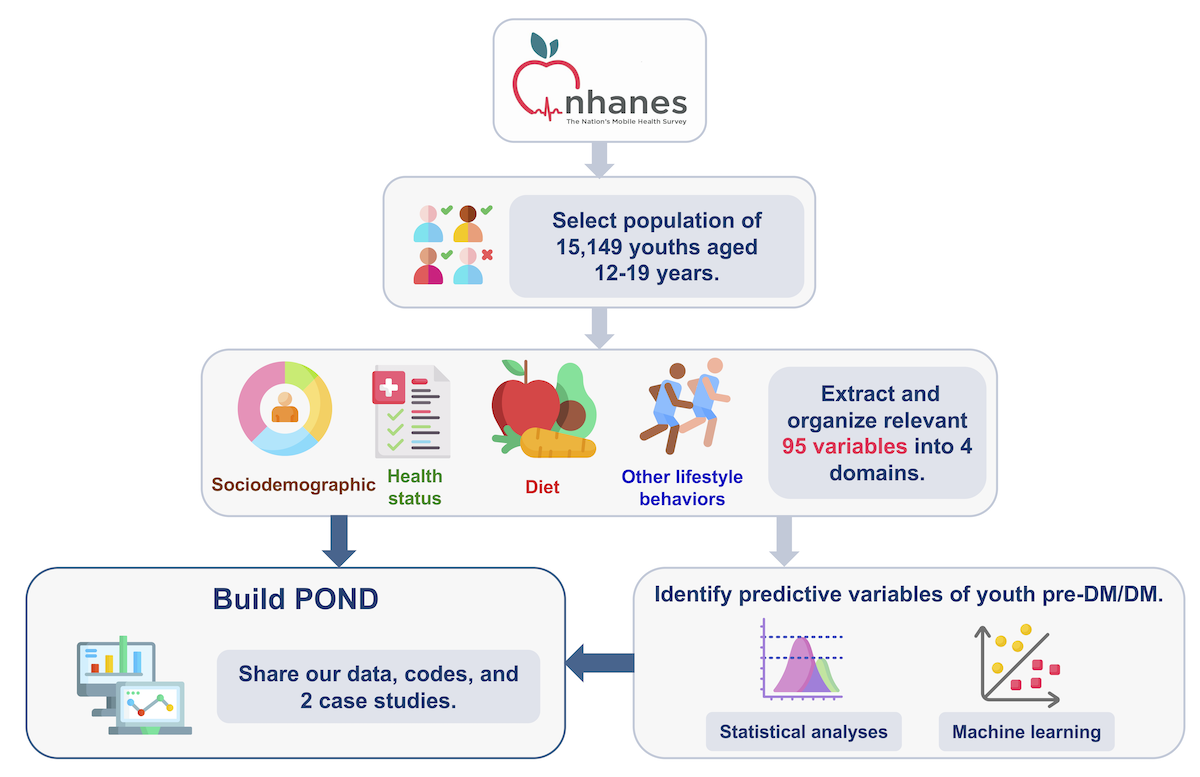
Study design and workflow. We processed data from 10 survey cycles (1999-2018) from the National Health and Nutrition Examination Survey (NHANES), which yielded 15,149 youths with known pre-DM/DM status. We extracted 95 variables that were relevant to pre-DM/DM and organized them into 4 domains: sociodemographic, health status, diet, and other lifestyle behaviors. We made the data set easily accessible to the public through the user-friendly POND (Prediabetes/diabetes in youth Online Dashboard) web portal, enabling users to navigate, visualize, and download the data. In addition, we conducted 2 case studies with complementary statistical and machine learning methods that are designed to illustrate the translation potential of our data set and point. Both analyses identified predictive variables associated with youth diabetes, and the results can be explored in POND (some images in this figure were obtained from an open-source collection). DM: diabetes mellitus.

Figure 1 [48] shows the overall study design and workflow. In the following subsections, we detail the components of the workflow.

### Data Source and Study Population

We built the youth pre-DM/DM data set based on publicly available NHANES data [49] spanning the years from 1999 to 2018. Developed by the Centers for Disease Control and Prevention, NHANES is a serial cross-sectional survey that gathers comprehensive health-related information from nationally representative samples of the noninstitutionalized population in the United States. The survey uses a multistage probability sampling method and collects data through questionnaires, physical examinations, and biomarker analysis. Each year, approximately 5000 individuals are included in the survey, and the data are publicly released in 2-year cycles.

**Figure 2:**
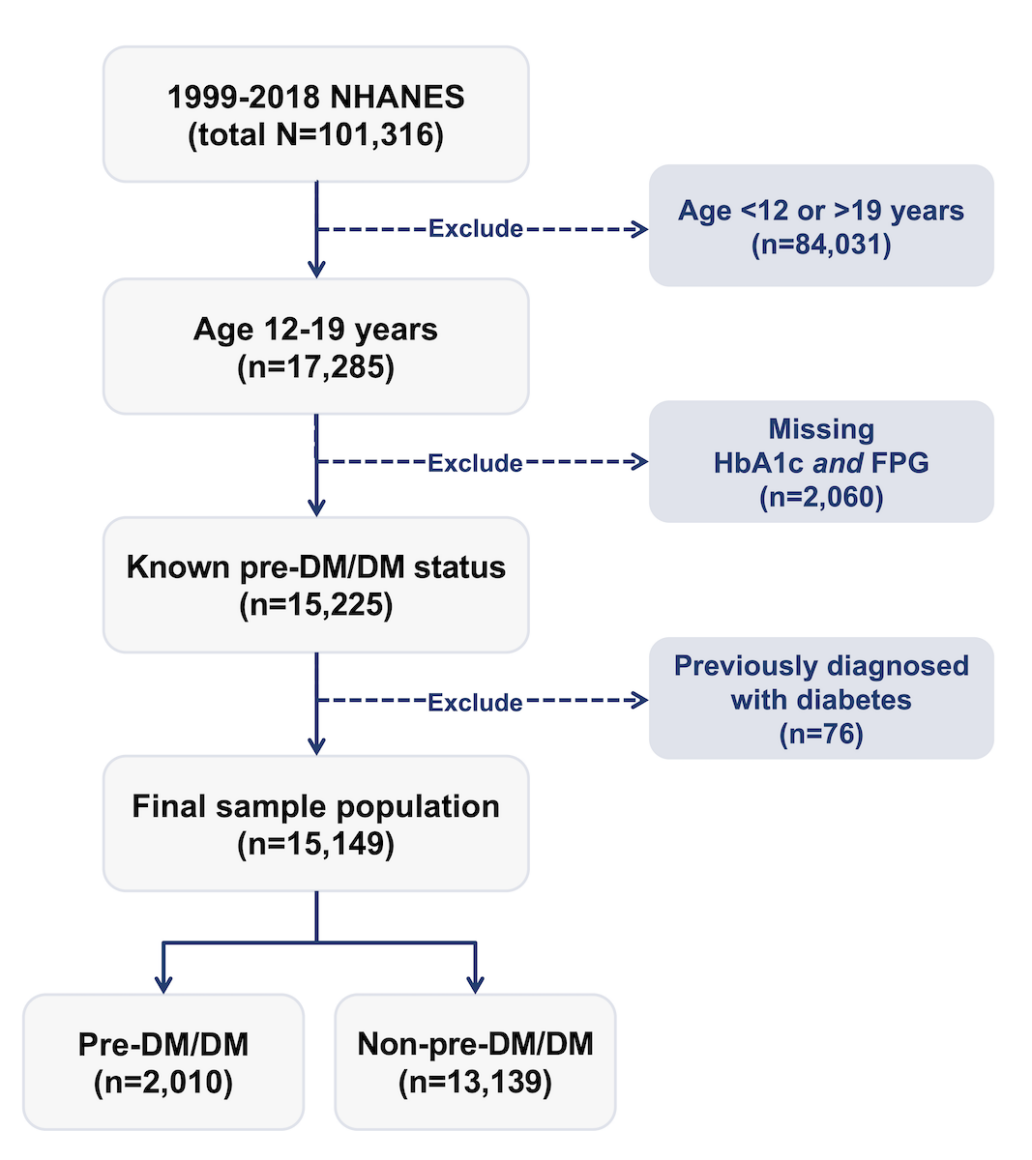
Flow chart showing the inclusion and exclusion criteria applied to 1999-2018 NHANES participants that yielded the study population included in our youth pre-DM/DM data set. Pre-DM/DM status was defined by the current American Diabetes Association (ADA) biomarker criteria, that is, elevated levels of 1 of 2 pre-DM/DM biomarkers (FPG ≥100 mg/dL or HbA1c ≥5.7%). DM: diabetes mellitus; FPG: fasting plasma glucose; HbA1c: glycated hemoglobin; NHANES: National Health and Nutrition Examination Survey.

Figure 2 details the process used to define our study population. Briefly, of the total 101,316 participants in 1999-2018 NHANES, we excluded individuals who (1) were not within the 12-19 years age range, (2) did not have either of the biomarkers used to define pre-DM/DM status, and (3) answered “Yes” to “Have you ever been told by a doctor or health professional that you have diabetes?” The youth pre-DM/DM outcome of this work was derived as follows: youth were considered at risk of pre-DM/DM if their fasting plasma glucose (FPG) was at or greater than 100 mg/dL, or their glycated hemoglobin (HbA_1c_) was at or greater than 5.7%, according to the current American Diabetes Association (ADA) pediatric clinical guidelines [2].

### Validation of the Study Population

We estimated pre-DM/DM prevalence across the 10 survey cycles (1999-2018) by incorporating the NHANES design elements in the analysis and compared the general trend with those reported in the literature [18,19]. We also specifically applied the analytical methods reported in a recent study [13] based on NHANES data to our study population to replicate the trends in pre-DM among youth in the United States from 1999 to 2018 reported in that analysis. Specifically, that study selected a youth population from 12-19 years of age with positive sampling weight from the fasting subsample (ie, nonzero and nonmissing Fasting Subsample 2 Year Mobile Examination Centers Weight [“WTSAF2YR”]; personal communication) without a self-reported physician-diagnosed DM. In addition, that study focused only on pre-DM, which was defined as an HbA_1c_ level between 5.7% and 6.4% or an FPG level between 100 mg/dL and 125 mg/dL [13].

### Development of Youth Pre-DM/DM Data Set

Based on the most recent ADA standard of care recommendations including factors related to pre-DM/DM risk and management [2], we selected 27 potentially relevant NHANES questionnaires and grouped them into 4 domains: sociodemographic, health status, diet, and other lifestyle behaviors. For example, under the health status domain, BMI was included as a potential risk factor for youth pre-DM/DM [2]. Similarly, lifestyle and behavioral variables included factors, such as diet and physical activity, that have been shown to be critical for pre-DM/DM prevention in both observational studies and randomized clinical trials [50-52]. Our sociodemographic domain included demographic, socioeconomic, and SDoH variables (eg, age, gender, poverty status, and food security). Except for commonly available clinical measurements, such as blood pressure and total cholesterol, we did not include laboratory data (eg, triglycerides, transferrin, C-reactive protein, interleukin-6, and white blood cells), since these measurements were not collected for all NHANES participants and were not commonly accessible for the general population.

From the selected questionnaires, we identified a list of 95 variables based on the aforementioned methodology. The complete list of variables is provided in Table S1 in Section S1 of Multimedia Appendix 1 [13,49,53-62] and on our POND web portal [47]. All the code developed, processed data, and detailed description of variables are also available on the web portal [47]. The process of extracting these variables involved extensive examination of the questions that were asked, consultation of the literature, and discussions to reach consensus within the study team. The details of this process are provided in Figure S1 and Section S2 of Multimedia Appendix 1. We used SAS (version 9.4; SAS Institute) and R (version 4.2.2; R Core Team, 2022) in R Studio (version 4.2.2; R Core Team, 2022) for data processing and data set development.

### Building the POND

To facilitate other researchers’ use of our youth pre-DM/DM data set and make our methodology transparent and reproducible, we developed POND to share our processed data set and enable users to understand and explore the data on their own. The web portal was developed using R *markdown* and the *flexdashboard* package [63] and was published as a Shiny application [64]. Table S2 and Section S3 in Multimedia Appendix 1 provide details of all the R packages used to develop POND, and the related code is available on the portal’s download page.

### Case Studies in Using the Data Set to Better Understand Youth Pre-DM/DM

#### Overview

To examine the validity and use of our data set for advancing translational research on youth pre-DM/DM, we conducted 2 complementary data analyses. We first conducted bivariate analyses to assess the statistical associations between each of the 95 variables and youth pre-DM/DM status. In the second analysis, we used ML methods to examine the ability to predict pre-DM/DM status of youth based on the 95 variables. The methodological details of these analyses are provided in the following subsections.

#### Bivariate Analyses to Identify Variables Associated With Pre-DM/DM Status

We examined associations between individual variables and youth pre-DM/DM status using chi-square and Wilcoxon rank sum tests for categorical and continuous variables, respectively. Cell sizes were checked for sufficient size (≥5) prior to chi-square tests. Independence and equal variance were assessed for continuous variables. Distribution normality was ensured through adequate sample size in accordance with Central Limit Theorem [65]. We applied Bonferroni correction for multiple hypothesis testing (n=95 tests) at an α level of .05 to determine the statistical significance of each association at the adjusted α level of .0005 (ie, approximately 0.05/95). We used Cramer V and Wilcoxon R values [66] as the effect size measures for categorical and continuous variables, respectively. To better compare with results from the ML approach, the main bivariate analyses did not account for NHANES survey design; thus, the results were applicable only to the study population included in the analytical sample and were not generalizable to the entire US youth population. For completeness, we provide the survey-weighted analyses using NHANES examination weights (“WTMEC2YR”) in Section S4 of Multimedia Appendix 1.

#### Prediction of Pre-DM/DM Status Using ML Algorithms

Several ML algorithms have been used to predict adult pre-DM/DM status using NHANES data [67-69], and we have previously used these algorithms to predict pre-DM/DM status specifically among youth in a subsample of our current study population [42]. We expanded these existing analyses by taking into account the multidomain nature of our data set with the goal of building an effective and interpretable predictive model of youth pre-DM/DM. To that end, we leveraged our recently developed ML framework, Ensemble Integration (EI) [53,54], with all 4 domains and their variables in our data set. EI incorporates both consensus and complementarity in our data set by first inferring local predictive models from the individual domains, that is, sociodemographic, health status, diet, and other lifestyle behaviors, that are expected to capture information and interactions specific to the domains. These local models and information are then integrated into a global pre-DM/DM, comprehensive pre-DM/DM prediction model using heterogeneous ensemble algorithms [70] (Figure S2, Table S3, and Table S4 under Section S5 in Multimedia Appendix 1). These algorithms, such as stacking, allow the integration of an unrestricted number and variety of local models into the global predictive model, thus offering improved performance and robustness. EI also enables the identification of the most predictive variables in the final model, thus offering deeper insights into the outcome being predicted.

We used both the aforementioned capabilities of EI to build and interpret a predictive model of youth pre-DM/DM status based on our data set. We also compared the predictive performance of the model with three alternative approaches: (1) a modified form of the ADA screening guideline [55], which is based on BMI, total cholesterol level, hypertension, and race or ethnicity, to assess the use of data-driven screening for youth pre-DM/DM (Table S5 in Multimedia Appendix 1); (2) EI applied to individual variable domains, namely, sociodemographic, health status, diet and other lifestyle behaviors, to assess the value of multidomain data for youth pre-DM/DM prediction; and (3) extreme gradient boosting (XGBoost) [71] applied to our combined multidomain data set as a representative alternate ML algorithm. This alternative was chosen as XGBoost is considered the most effective classification algorithm for tabular data [72], since it can potentially capture feature interactions across different domains [73,74]. The prediction performance of EI and all the alternative approaches were assessed in terms of the commonly used area under the receiver operating characteristic curve (AUROC) [75] and balanced accuracy (BA; average of specificity and sensitivity) [76] measures. The performance of the ML-based prediction approaches, namely, multi- and single-domain EI and XGBoost, was evaluated in a 5-fold cross-validation setting repeated 10 times [77]. These performance scores were statistically compared using the Wilcoxon rank sum test, and the resultant *P* values were corrected for multiple hypothesis testing using the Benjamini-Hochberg procedure to yield false discovery rates (FDRs) [78]. More details of ML model building; the alternative approaches; and the evaluation methodology, including cross-validation, model selection, and comparison, are available in section S5 in Multimedia Appendix 1. Finally, we used EI’s interpretation capabilities [53,54] to identify the variables in our data set that were the most predictive of youth pre-DM/DM status and compare them with the variables identified from the bivariate analyses described in the above subsection.

### Ethical Considerations

This study used existing deidentified and anonymized data in the public domain directly downloadable from the NHANES website and thus, according to the Common Rule, was exempt from institutional review board review and the informed consent requirement. NHANS was conducted by the Centers for Disease Control and Prevention National Center for Health Statistics. NHANES survey procedures and protocol were approved by the National Center for Health Statistics ethics review board for each survey cycle [79].

## Results

### Study Population Derived From NHANES

Our study population consisted of 15,149 youths aged 12-19 years who participated in the 1999-2018 NHANES cycles and met our selection criteria (Figure 2). Approximately 13.3% (2010/15,149) of US youth were at risk of pre-DM/DM according to the clinically standard criteria for defining pre-DM/DM per ADA guidelines (FPG ≥100 mg/dL and HbA_1c_ ≥5.7%; Table 1).

**Table 1:** Unweighted study population characteristics^a^.

Variables		Overall (N=15,149)	With pre-DM/DM^b^ (n=2010; unweighted %=13.3)^b^	With no pre-DM/DM (n=13,139)
**Sociodemographic**				
	Age (years), median (IQR)	15 (13-17)	15 (13-17)	16 (14-17)
	Female sex, n (%)	7430 (49)	691 (34.4)	6739 (51.3)
**Race or ethnicity, n (%)**				
	Black, non-Hispanic	4292 (28.3)	676 (33.6)	3616 (27.5)
	Hispanic	5565 (36.7)	711 (35.4)	4854 (36.9)
	White, non-Hispanic	4033 (26.6)	431 (21.4)	3602 (27.4)
	Other	1259 (8.3)	192 (9.6)	1067 (8.1)
**Insurance, n (%)**				
	Private	6392 (43)	744 (37.7)	5648 (43.8)
	Medicare, government, or single service	2026 (13.6)	268 (13.6)	1758 (13.6)
	Medicaid or CHIP^c^	3637 (24.4)	564 (28.6)	3073 (23.8)
	No insurance	2821 (19)	395 (20)	2426 (18.8)
	Authorized for food stamps	7833 (69.4)	1037 (61.1)	6796 (70.8)
**Health status**				
	BMI percentile, n (%)			
	Underweight (BMI percentile < 5th), n (%)	462 (3.1)	40 (2.0)	422 (3.2)
	Normal weight (5th ≤ BMI percentile < 85th), n (%)	8516 (56.8)	933 (46.8)	7583 (58.4)
	Overweight (85th ≤ BMI percentile < 95th), n (%)	2788 (18.6)	356 (17.9)	2432 (18.7)
	Obese (95th ≤ BMI percentile), n (%)	3214 (21.5)	663 (33.3)	2551 (19.6)
	Hypertensive^d^, n (%)	2552 (17.4)	502 (26.1)	2050 (16.1)
	High total cholesterol (≥170 mg/dL), n (%)	4951 (33.2)	707 (35.6)	4244 (32.8)
	Fasting plasma glucose (mg/dL), median (IQR)	93 (88-98)	102 (100-106)	91 (86-95)
	Hemoglobin A_1c_ (%), median (IQR)	5.2 (5.0-5.4)	5.5 (5.2-5.7)	5.2 (5.0-5.3)
**Diet, median (IQR)**				
	Meals eaten out per week	2 (1-3)	2 (1-3)	2 (1-3)
	Total grain (oz eq^e^) intake 24 hours prior	6.55 (4.24-9.66)	6.43 (4.19-9.58)	6.57 (4.25-9.67)
	Total fruits (cup eq) intake 24 hours prior	0.38 (0.00-1.44)	0.26 (0.00-1.37)	0.40 (0.00-1.45)
	Total vegetable (cup eq) intake 24 hours prior	0.88 (0.39-1.58)	0.84 (0.37-1.54)	0.89 (0.39-1.59)
	Total protein (oz eq) intake 24 hours prior	5.29 (2.71-9.15)	4.73 (2.46-8.37)	5.38 (2.76-9.34)
	Added sugar (tsp eq) intake 24 hours prior	20.42 (11.49-32.49)	20.09 (11.15-31.89)	20.48 (11.57-32.59)
**Other lifestyle behavior**				
	Physical activity minutes per week, median (IQR)	209 (45-488)	210 (49-476)	209 (45-491)
	Screen time hours per day, median (IQR)	5 (3-8)	5 (3-8)	5 (2-7)
	Exposed to secondhand smoke at home, n (%)	3297 (21.9)	469 (23.6)	2828 (21.7)

^a^Unweighted statistics of some key variables describing the study population in the youth pre-DM/DM data set overall and by pre-DM/DM status. More detailed statistics for all the variables in our data set can be found in the Data Exploration section of POND.

^b^Pre-DM/DM: pre–diabetes mellitus and diabetes mellitus.

^c^CHIP: child health insurance program.

^d^Hypertensive was defined by blood pressure ≥90th percentile or ≥120/80 mm Hg for children 13 years of age and older [2].

^e^eq: equivalent.

### Validation of the Study Population

We estimated that the survey-weighted prevalence of pre-DM/DM in our study population rose substantially from 4.1% (95% CI 2.8-5.4) in 1999 to 22% (95% CI 18.5-25.6) in 2018 (Figure S3 and section S6 in Multimedia Appendix 1). This increasing trend of pre-DM/DM prevalence was consistent with that reported in other NHANES-based studies, which had pre-DM/DM prevalence ranging from 17.7% to 18% [18,19]. We also applied the study population and pre-DM definition criteria reported in a recent study [13] to NHANES data and derived a similarly sized study population (n=6656 vs n=6598 in the current vs previous analysis [13]) and youth pre-DM prevalence, which ranged from 11.1% (95% CI 8.9-13.3) to 37.3% (95% CI 31.0-43.6) in our analysis compared with from 11.6% (95% CI 9.5-14.1) to 28.2% (95% CI 23.3-33.6) in the study by Liu et al [13] (Table S6 in Multimedia Appendix 1).

### Youth Pre-DM/DM-Focused Data Set

We extracted 95 epidemiological variables from NHANES and organized them into 4 pre-DM/DM-related domains, namely, sociodemographic, health status, diet, and other lifestyle behaviors (Table S1 in Multimedia Appendix 1). Table 1 shows the unweighted statistics of some key study population characteristics. Among youth with pre-DM/DM (n=2010), the proportion of youth who were non-Hispanic Black, non-Hispanic White, Hispanic, and other race or ethnicity (including non-Hispanic persons who reported races other than Black or White and non-Hispanic Asian) were 33.6% (n=676), 21.4% (n=431), 35.4% (n=711), and 9.6% (n=192), respectively. Approximately, half (7719/15,149, 51%) of the population were male, and they represented 65.6% (1319/2010) of those with pre-DM/DM. Approximately 32.4% (4528/15,149) of the youth had a family income below poverty level, and 69.4% (7833/15,149) were from households receiving food stamps. The proportion of youth covered by private insurance was higher among those with than with no pre-DM/DM (5648/13,139, 43.8% vs 744/2010, 37.7%). Overall, 21.5% (3214/15,149) of the youth were obese as defined by having a BMI at or above the 95th percentile based on age and gender, and the proportion was 33.3% (663/2010) among youth with pre-DM/DM. Youth with pre-DM/DM tended to have less fruit and vegetable intake and ate lower amounts of protein and total grains than those with no pre-DM/DM. Youth with and with no pre-DM/DM showed similar amounts of physical activity with 209 and 210 minutes per week, respectively (Table 1).

### Pre-DM/DM in Youth Online Dashboard

**Figure 3:**
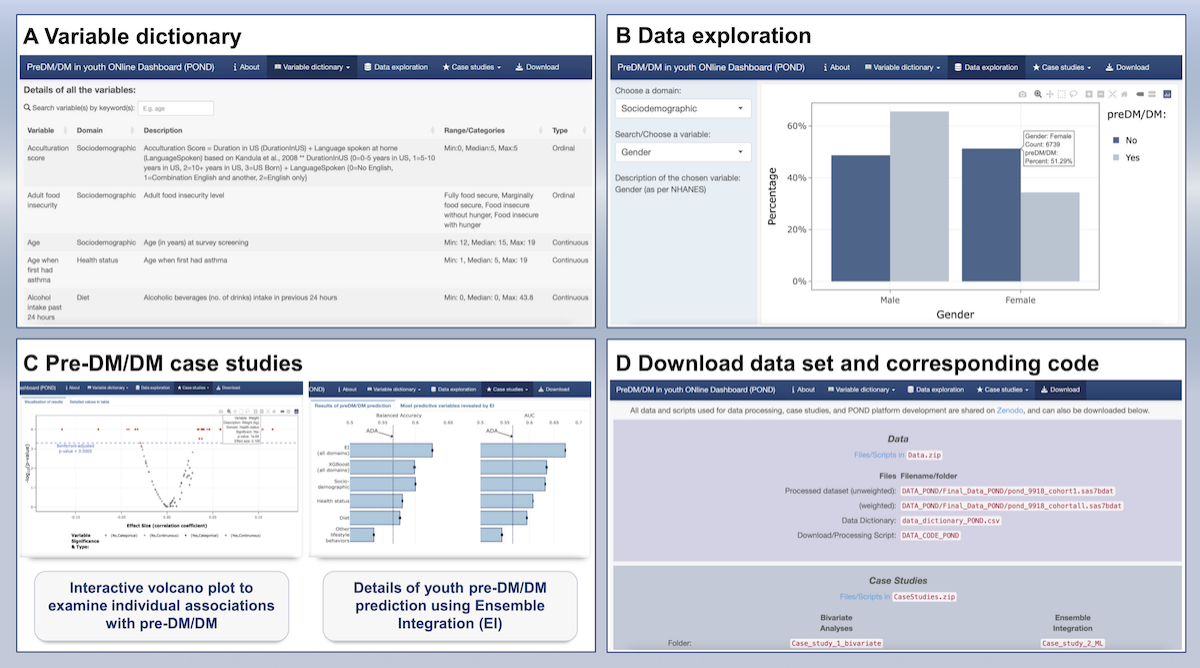
Screenshots of different functionalities available in POND (Prediabetes/diabetes in youth Online Dashboard). (A) Detailed dictionary of the 95 variables included in our youth pre-DM/DM database organized by 4 domains. (B) Data exploration section showing the distribution of user-selectable variables by pre-DM/DM status. (C) Case study section detailing the results of bivariate association analyses and the prediction of youth pre-DM/DM status from machine learning approaches. (D) Download section, where the data set and the code used in this study are publicly available to facilitate reproducibility and further exploration for interested users. ADA: American Diabetes Association; DM: diabetes mellitus; NHANES: National Health and Nutrition Examination Survey.

To facilitate other researchers’ use of our youth pre-DM/DM data set and make our methodology transparent and reproducible, we developed POND, which is available on [47]. Users can navigate POND through its built-in functionalities. For example, users are able to explore the details of the 95 individual variables (Figure 3A) and their distributions by pre-DM/DM status (Figure 3B), examine the risk factors of youth pre-DM/DM identified from the case studies described below (Figure 3C), as well as download the data for customized analysis and the analytical code to replicate our findings (Figure 3D). In addition, we make available all the code used to develop the data set, our case studies, and POND itself.

### Case Studies Using Our Data Set to Better Understand Youth Pre-DM/DM

#### Overview

We examined the validity and use of our processed multidomain data set for translational studies on youth pre-DM/DM by the following 2 complementary types of data analyses.

#### Identifying Individual Variables Associated With Pre-DM/DM Status

**Figure 4:**
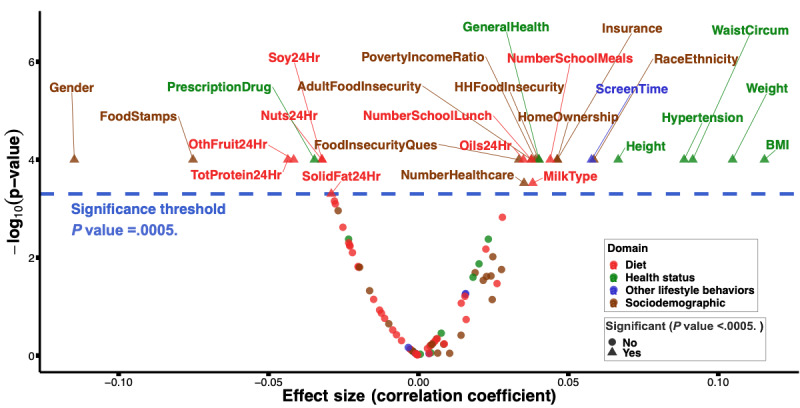
Individual variables associated with youth pre-DM/DM status based on bivariate analyses. This volcano plot shows the *P* values and the effect sizes of the associations between the individual variables and youth pre-DM/DM status. Categorical and continuous variables were tested for association using chi-square and Wilcoxon rank sum tests, respectively. The effect size was measured by Cramer V for categorical variables and Wilcoxon r value for continuous ones. After Bonferroni adjustment for multiple hypothesis testing, we found 27 variables to be significantly (*P*<.001; blue dotted line) associated with youth pre-DM/DM status. These are named above the blue dotted line in this plot and colored by the domain they belong to. DM: diabetes mellitus; HH: household.

In our bivariate analyses, we found 27 variables to be significantly (*P*<.001, Bonferroni adjusted) associated with pre-DM/DM status (Figure 4 [63] and Table S7 in Multimedia Appendix 1). These variables spanned all 4 domains and included gender, race or ethnicity, use of food stamps, health insurance status, BMI, total protein intake, and screen time. Similar results were found when repeating these bivariate association tests after accounting for NHANES survey design elements (Table S7 in Multimedia Appendix 1).

#### Predicting Youth Pre-DM/DM Status With ML

We used an ML framework, EI [53,54], to leverage the multidomain nature of our data set and predict youth pre-DM/DM status. We also compared EI’s performance with alternative prediction approaches, most prominently the widely used XGBoost algorithm [71].

**Figure 5:**
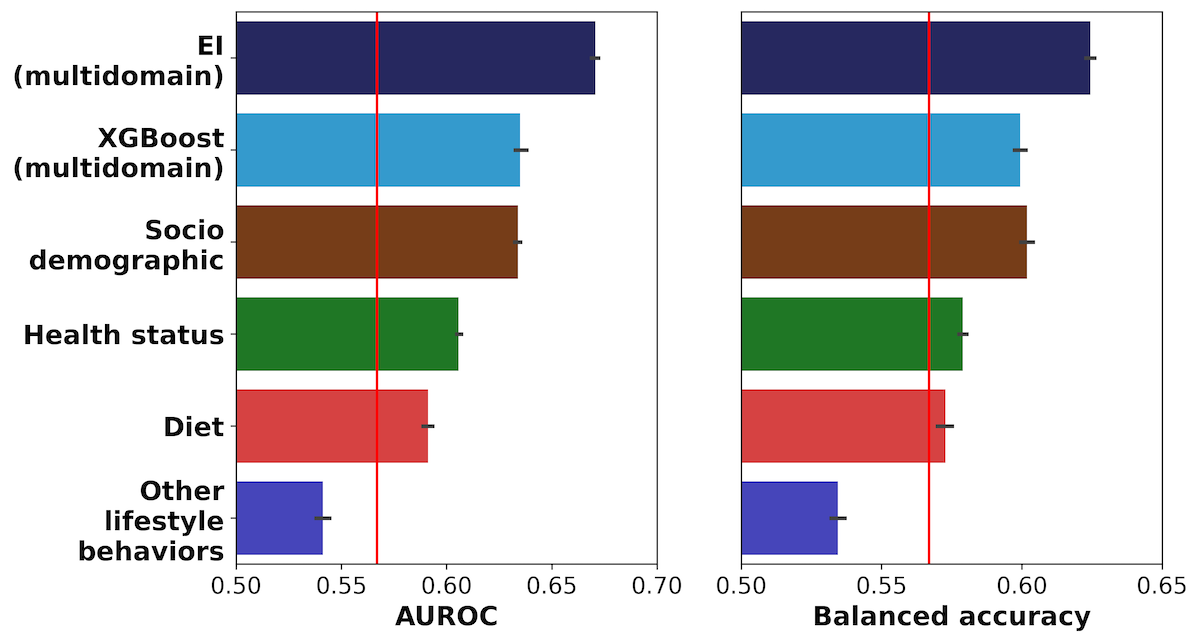
Comparison of the performance of multiple approaches for predicting youth pre-DM/DM status based on machine learning approaches. We compared the performance of the multidomain Ensemble Integration (EI) approach with 3 alternative prediction approaches. The alternative approaches were (1) a modified form of the American Diabetes Association (ADA) screening guideline (vertical red line), (2) single-domain EI-based prediction based on each of the 4 individual domains, and (3) the commonly used extreme gradient boosting (XGBoost) algorithm applied to our whole data set. Performance was measured in terms of the area under the receiver operating characteristic curve and balanced accuracy (average of sensitivity and specificity) measures. For each machine learning approach, the horizontal bar shows the average of the corresponding scores and the error bar indicates the corresponding standard error measured over 10 rounds of 5-fold cross-validation. AUROC: area under the receiver operating characteristic curve; EI: Ensemble Integration.

The best-performing multidomain EI methodology, stacking [75] using logistic regression, predicted youth pre-DM/DM status (AUROC=0.67; BA=0.62) more accurately than all the alternative approaches (Figure 5), namely, XGBoost (AUROC=0.64; BA=0.60; Wilcoxon rank sum FDR=1.7×10^-4^ and 1.8×10^-4^, respectively), the ADA pediatric screening guidelines (AUROC=0.57, BA=0.57; Wilcoxon rank sum FDR=1.7×10^-4^ and 1.8×10^-4^, respectively), and 4 single-domain EI (AUROC=0.63-0.54; BA=0.60-0.53; FDR <1.7×10^-4^ and 1.8×10^-4^, respectively).

**Figure 6:**
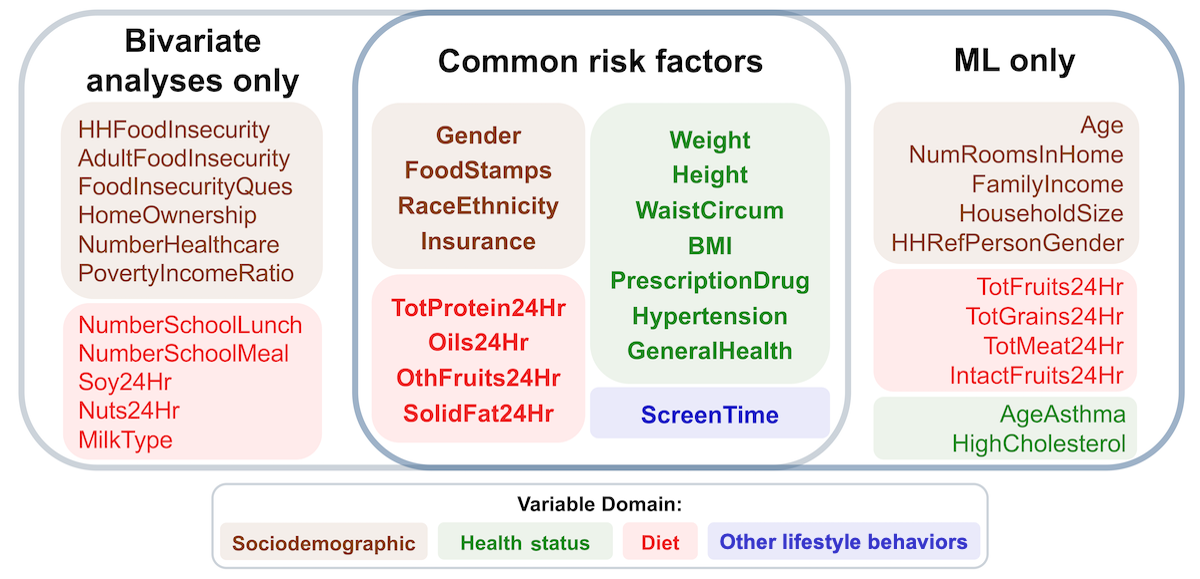
Variables associated with youth pre-DM/DM selected by bivariate analyses and the multidomain Ensemble Integration (EI) approaches. Venn diagram summarizing the overlap between the 27 significant variables identified in the bivariate analyses and the 27 most predictive variables identified from the multidomain EI model. We found that 16 variables overlapped between the 2 methods (Fisher *P*=7.06×10–6) and were drawn from all 4 domains (shown in different colors), indicating the multifactorial nature of youth pre-DM/DM. DM: diabetes mellitus; HH: household; ML: machine learning.

The multidomain EI also identified 27 variables (the same as the number of significant variables from bivariate analyses) that contributed the most to predicting youth pre-DM/DM status. Among these variables, 16 overlapped with those identified from the bivariate statistical analyses (Figure 6; Fisher *P* of overlap=7.06×10^-6^). These variables identified by both approaches included some established pre-DM/DM risk factors such as BMI and high total cholesterol, as well as some less-recognized ones such as screen time and taking prescription drugs [2].

## Discussion

### Principal Findings

Leveraging the rich information in NHANES spanning nearly 20 years, we built the most comprehensive epidemiological data set for studying youth pre-DM/DM. We accomplished this by selecting and harmonizing variables relevant to youth pre-DM/DM from sociodemographic, health status, diet, and other lifestyle behaviors domains. This youth pre-DM/DM data set, as well as several functionalities to explore and analyze it, is publicly available in our user-friendly web portal, POND. We also conducted case studies using the data set with both traditional statistical methods and ML approaches to demonstrate the potential of using this data set to identify factors relevant to youth pre-DM/DM. The combination of the comprehensive public data set and POND provides avenues for more informed investigations of youth pre-DM/DM.

The future translational impact of pre-DM/DM research, facilitated by comprehensive data sets such as the one developed in this study, holds significant promise for advancing our understanding of the disease and its risk factors among youth. By enabling researchers to investigate multifactorial variables associated with pre-DM/DM, this data set contributes to several areas of research and has a broader impact on the scientific community. First, the data set’s comprehensive nature allows researchers to explore the collective impact of various risk factors across multiple health domains. By incorporating sociodemographic factors, health status indicators, diet, and lifestyle behaviors, researchers can gain a holistic understanding of the interplay between these factors and pre-DM/DM risk among youth. This knowledge can be used to generate hypotheses for further studies and inform the development of targeted interventions and prevention strategies that address the specific needs of at-risk populations. Furthermore, the data set provides an opportunity to delve into less-studied variables and their interactions in relation to pre-DM/DM risk. Variables such as screen time, acculturation, or frequency of eating out, which are often overlooked in traditional research, can be examined to uncover their potential influence on pre-DM/DM risk among youth. This expands the scope of translational research and enhances our understanding of the multifaceted nature of the disease.

One of the major contributions of our work was POND, our publicly available web portal, which provided access to all materials related to our data set and analyses, thus enabling transparency and reproducibility. Although several such portals are available in other biomedical areas, such as genomics [76-78], there is a general lack of such tools in epidemiology and public health. We hope that, in addition to facilitating studies into pre-DM/DM, POND illustrates the use of such portals for population and epidemiological studies as well.

The results of the case studies and validation exercises we conducted were also consistent with existing literature. The case studies identified known pre-DM/DM risk factors, such as gender [15,17,19], race and ethnicity [2,9,10,24], health measures (BMI, hypertension, and cholesterol) [2,55], income [9,11], insurance status [9,10], and health care availability [9,10], thus affirming the validity of the data set. In addition, our analyses revealed some less studied variables, such as screen time, home ownership status, self-reported health status, soy and nut consumption, and frequency of school meal intake, which may influence youth pre-DM/DM risk. Further study of these variables may reveal new knowledge about pre-DM/DM among youth. More generally, such novel findings further demonstrate the use of our data set and data-driven methods for further translational discoveries about this complex disorder.

### Limitations

Although our work has several strengths and high potential use for youth pre-DM/DM studies, it is not without limitations. First, as our data set was derived from NHANES, we adopt limitations to the survey in our data set. Since NHANES is a cross-sectional survey, the pre-DM/DM status and its related variables provide only consecutive snapshots of youth in the United States over time across the available survey cycles. Thus, the associations identified are better suited for hypothesis generation purposes and require in-depth investigation using prospective longitudinal and randomized trial designs. In addition, we modified the ADA guideline for determining pre-DM/DM status according to variable availability. Due to the high missingness of 45% in family history (DIQ170) and the complete missingness of maternal history (DIQ175S) from 1999 to 2010 in the raw NHANES data, we were unable to include family history of diabetes in the data set. Similarly, NHANES does not provide data regarding every condition associated with insulin resistance. Therefore, we used hypertension and high cholesterol as proxies for insulin resistance. On the other hand, as our main purpose is to use POND as a conduit between this comprehensive youth pre-DM/DM database and interested researchers, our method can be adopted to longitudinal data sets should they become available in the future. Second, for the prediction of pre-DM/DM status, EI’s performance was found to be significantly better than the alternative approaches, including a modified form of the suggested guideline [45]. However, this performance assessment was based only on cross-validation, which is no substitute for validation on external data sets that is necessary for rigorous assessment. Finally, while our preliminary case study analyses identified a wide range of variables associated with youth prediabetes and diabetes, other known risk factors, such as current asthma status [80-82], added sugar consumption [83-85], sugary fruit and juice intake [83-86], and physical activity per week [6-8,50], were not identified. This limitation can be addressed by using other data analysis methods beyond our bivariate testing and ML approaches, highlighting more potential use cases of our data set.

### Conclusions

Overall, the future impact of translational pre-DM/DM research facilitated by comprehensive data sets and web servers like ours extends beyond individual studies. It creates opportunities for interdisciplinary collaboration and reproducibility, strengthens evidence-based decision-making, and supports the development of targeted interventions for the prevention and management of pre-DM/DM among youth. By providing rich resources, our work can enable researchers to build upon existing knowledge and push the boundaries of translational pre-DM/DM research, ultimately leading to improved health outcomes for at-risk populations.

## Appendix

Multimedia Appendix 1 Supplemental materials.

## Abbreviations

ADA: American Diabetes Association
AUROC: area under the receiver operating characteristic curve
BA: balanced accuracy
DM: diabetes mellitus
EI: Ensemble Integration
FDR: false discovery rate
FPG: fasting plasma glucose
HbA1c: glycated hemoglobin
ML: machine learning
NHANES: National Health and Nutrition Examination Survey
POND: Prediabetes/diabetes in youth Online Dashboard
pre-DM: pre–diabetes
SDoH: social determinants of health
XGBoost: extreme gradient boosting

## Data Availability

The data set and code used in this study are available at Zenodo [87] and our web portal POND [47].
