# Correction: A Comprehensive Youth Diabetes Epidemiological Data Set and Web Portal: Resource Development and Case Studies

**DOI:** 10.2196/69136

**Published:** 2024-11-27

**Authors:** Catherine McDonough, Yan Chak Li, Nita Vangeepuram, Bian Liu, Gaurav Pandey

**Affiliations:** 1 Department of Genetics and Genomic Sciences Icahn School of Medicine at Mount Sinai New York, NY United States; 2 Department of Pediatrics Icahn School of Medicine at Mount Sinai New York, NY United States; 3 Department of Population Health Science and Policy Icahn School of Medicine at Mount Sinai New York, NY United States

In "A Comprehensive Youth Diabetes Epidemiological Data Set and Web Portal: Resource Development and Case Studies" (JMIR Public Health Surveill 2024;10:e53330) a few errors were noted:

1) In the Abstract, under "Results", the following sentence:

Among these factors, 16 factors were also identified based on the EI methodology (Fisher *P* of overlap=7.06×10^6^).

has been revised to include the minus symbol in the superscript of the *P* value, as follows:

Among these factors, 16 factors were also identified based on the EI methodology (Fisher *P* of overlap=7.06×10^-^^6^).

2) In "Predicting Youth Pre-DM/DM Status With ML", in the Results section, the following excerpt:

…Wilcoxon rank sum FDR=1.7×10^4^ and 1.8×10^4^, respectively), the ADA pediatric screening guidelines (AUROC=0.57, BA=0.57; Wilcoxon rank sum FDR=1.7×10^4^ and 1.8×10^4^, respectively), and 4 single-domain EI (AUROC=0.63-0.54; BA=0.60-0.53; FDR <1.7×10^4^ and 1.8×10^4^, respectively).

has been revised to include the minus symbol in the superscripts of the false discovery rates (FDR), as follows:

…Wilcoxon rank sum FDR=1.7×10^-^^4^ and 1.8×10^-^^4^, respectively), the ADA pediatric screening guidelines (AUROC=0.57, BA=0.57; Wilcoxon rank sum FDR=1.7×10^-^^4^ and 1.8×10^-^^4^, respectively), and 4 single-domain EI (AUROC=0.63-0.54; BA=0.60-0.53; FDR <1.7×10^-^^4^ and 1.8×10^-^^4^, respectively).

3) Also in "Predicting Youth Pre-DM/DM Status With ML", in the Results section, the following sentence:

Among these variables, 16 overlapped with those identified from the bivariate statistical analyses (Figure 6; Fisher *P* of overlap=7.06×10^6^)

has been revised to include the minus symbol in the superscript of the *P* value, as follows:

Among these variables, 16 overlapped with those identified from the bivariate statistical analyses (Figure 6; Fisher *P* of overlap=7.06×10^-^^6^).

The correction will appear in the online version of the paper on the JMIR Publications website on November 27, 2024 together with the publication of this correction notice. Because this was made after submission to PubMed, PubMed Central, and other full-text repositories, the corrected article has also been resubmitted to those repositories.

